# Noncovalent Interactions of Tiopronin-Protected Gold Nanoparticles with DNA: Two Methods to Quantify Free Energy of Binding

**DOI:** 10.1155/2014/143645

**Published:** 2014-01-22

**Authors:** R. Prado-Gotor, E. Grueso

**Affiliations:** Department of Physical Chemistry, Faculty of Chemistry, University of Sevilla, C/Profesor García González S/N, 41012 Sevilla, Spain

## Abstract

The binding of gold nanoparticles capped with N-(2-mercaptopropionyl)glycine (Au@tiopronin) with double-stranded DNA has been investigated and quantified in terms of free energies by using two different approaches. The first approach follows the DNA conformational changes induced by gold nanoparticles using the CD technique. The second methodology consists in the use of pyrene-1-carboxaldehyde as a fluorescent probe. This second procedure implies the determination of the “true” free energy of binding of the probe with DNA, after corrections through solubility measurements. Working at different salt concentrations, the nonelectrostatic and electrostatic components of the binding free energy have been separated. The results obtained revealed that the binding is of nonelectrostatic character, fundamentally. The procedure used in this work could be extended to quantify the binding affinity of other AuNPs/DNA systems.

## 1. Introduction

In recent years, the studies of noncovalent interactions of DNA with ligands have received considerable attention caused by the huge number of applications being derived from these interactions. Among these applications, the development of new diagnostic and therapeutic agents [1, 2], the exploration of the possibilities of DNA as molecular conductor [3], the interest in gene transport [4], and the fabrication of biosensors stand out [5–7]. In our group, we have a consistent background on the analysis of ligand-receptor interactions and of the kinetic analysis of DNA-containing systems [8, 9]. We considered (i) thermodynamic aspects of ligand-receptor (DNA) binding [10, 11]; (ii) the binding kinetics, including the influences of salts and cosolvents on the kinetics [12, 13], and (iii) the effect of DNA on the reactivity of ligands [14–16]. For these purposes, we have conveniently used substitution inert transition metal complexes [14], organic compounds [10, 12], surfactants [11, 12], bile salts [16], and gold nanoparticles [13].

Among a variety of these ligands, intense current interest is focused on gold colloids (AuNPs) due to their chemical stability, high biocompatibility and excellent structural, and optical, magnetic, and catalytic properties [17]. In particular, water soluble metal nanoparticles have received considerable attention due to the potential benefits in the fields of biology and medicine [18–22]. Especially interesting are alkanethiolates as protective agents due to their advantages of stability, suspendability in different solvents, and facile characterization by standard analytical techniques [23]. Since successful therapy for curing cancer and others genetic diseases requires the transport of DNA in the cell by delivery vehicles, the effective complexation of the DNA is a subject of great interest [24]. Numerous biological and medical applications in this active area of research are based on the binding of the nanoparticle probe to a particular biological substrate. More recently, sensors consisting of metal nanoparticles functionalized with DNA have appeared in the literature. It has been shown that these particles show affinities to the ligands that are, at least, two orders of magnitude greater than other conventional sensors [25–27]. However, little progress has been made in understanding noncovalent interactions of gold nanoparticles with nucleic acids [28–31] and quantitative studies on the affinity of these systems are even more scarce [29, 32, 33]. In this sense, the scope of this work is to quantify the thermodynamic aspects of DNA-Au@tiopronin binding. We are also interested in knowing how the environment surrounding the colloidal gold-DNA system, specifically the presence of salt (NaCl), affects the complex formation. The aim of this thermodynamic investigation carried out in conjunction with a complementary structural and spectroscopy study was to learn more about the factors that control the complexation of DNA with gold nanoparticles.

## 2. Materials and Methods

### 2.1. Chemicals and Reagents

All chemicals were Analytical R. Grade and were used without further purification. Hydrogen tetrachloroaurate(III) trihydrate and pyrene-1-carboxaldehyde (1-PyCHO) were purchased from Sigma-Aldrich; N-(2-mercaptopropionyl)glycine from Fluka; NaBH_4_ from Lancaster; and NaCl from Merck. Calf thymus DNA was purchased from Pharmacia (average number of base pairs: 10000) and used without further purification because preliminary experiments showed that purification does not produce any changes in experiments' results. Polynucleotide concentrations were determined spectrophotometrically from the molar absorptivity (6600 mol^−1^ dm^3^ cm^−1^ at 258 nm in order to have the DNA concentration in phosphate units) [34]. Solutions were prepared with deionized water, the conductivity being less than 10^−6^ S m^−1^. The temperature was maintained at 298.2 ± 0.1 in all experiments.

#### 2.1.1. Synthesis of Gold Nanoparticles (Au@tiopronin)

Au@tiopronin nanoparticles were prepared using the procedure of Templeton et al. [35]. One batch with hydrogen tetrachloroaurate(III) trihydrate (1 equiv) and N-(2-mercaptopropionyl)glycine (tiopronin) (5.5 equiv) was codissolved in 35 mL of 6 : 1 methanol/acetic acid, resulting in a ruby red solution. Sodium borohydride (22 equiv) in 15 mL of H_2_O was subsequently added via rapid stirring. The resultant brown suspension was stirred for an additional 30 min after cooling, with the solvent being removed under vacuum at 4°C. The crude sample was completely insoluble in methanol but reasonably soluble in water. It was purified by dialysis, in which the pH of the crude product dissolved in 20 mL of water (NANOpure) was adjusted to 1 by dropwise addition of concentrated hydrochloric acid. This solution was loaded into 15 cm segments of seamless cellulose ester dialysis membrane (Sigma, MWCO = 10.000), placed in 4 L beakers of water, and stirred slowly being recharged with fresh water ca. every 10 h over the course of 72 h. NMR spectra were used in order to see that the tiopronin was not free but bound to the gold. The dark brown Au@tiopronin solutions were collected from the dialysis tubes and were lyophilized. The product materials were found to be spectroscopically clean and produced a yield of 119 mg.

TEM analysis was carried out in a Philips CM 200 electron microscope working at 200 kV. Size distributions of the Au cores were measured from enlarged TEM image photographs for at least 200 individual cluster core images. A value of 1.4 nm was obtained for the diameter of the gold nanoparticle. Au@tiopronin nanoparticles were also characterized by visible absorption spectra and C, H, N, and S microanalysis (14.04% C; 2.25% H; 3.51% N; 7.78% S). According to these data and the results of the TEM, the relation between the number of Au atoms and tiopronin ligands was 119/105 [35].

All the experiments were carried out from solutions of gold nanoparticles prepared by weight.

### 2.2. UV-Vis Spectra

The spectra of the Au@tiopronin in the presence and in the absence of DNA were recorded with a Cary 500 spectrophotometer from 280 nm to 800 nm. The UV-vis absorption spectra showed a slight detectable surface plasmon band (SPB) as a consequence of the small size of the clusters (see Figure 2(a)). Titration experiments were carried out at a fixed colloidal gold concentration, [AuNPs] = 1.58 × 10^−6^ M, and in a DNA concentration range from 6.7 × 10^−6^ to 9.6 × 10^−5^ M.

### 2.3. Fluorescence Measurements

Fluorescence measurements were carried out in a spectrofluorimeter (Hitachi f-2500), interfaced to a PC for the reading and handling of the spectra. For the study with the fluorescence intercalator, pyrene-1-carboxaldehyde (1-PyCHO), intensity measurements were performed at [1-PyCHO] = 5.10^−7^ M. The excitation and emission wavelengths were 356 nm and 458 nm, respectively. It was checked that the results were independent of the excitation wavelength, provided that this one was in the range from 300 to 425 nm. DNA concentrations ranged from 10^−5^ M to 10^−3^ M. The water used in the preparation of solutions contained ethanol 5% (by weight). The presence of the alcohol was necessary in order to make the probe, pyren-1-carboxyaldehyde, soluble.

For AuNPs/DNA titrations, a fixed colloidal gold concentration of [AuNPs] = 1.5 × 10^−6^ M was used, the DNA concentration varying from 10^−5^ M to 7 × 10^−5^ M. The excitation wavelength was 451 nm whereas the emission wavelength was 656 nm. The validation of the Beer-Lambert law was checked in the range of [AuNPs] = 5 × 10^−8^–2.16 × 10^−6^ M.

### 2.4. Circular Dichroism (CD) Spectra

Electronic CD spectra were recorded in a BioLogic MOS-450 spectropolarimeter. A standard quartz cell of 10 mm path length was used. The spectra were expressed in terms of molar ellipticity. Scans were taken from 220 nm to 310 nm. For each spectrum, 5–10 runs were averaged with a 5 min equilibration before each scan. All the spectra were performed at a fixed concentration of [DNA] = 10^−4^ M.

### 2.5. Scanning Electron Microscopy (SEM)

Scanning electron microscopy (SEM) images were obtained by using a Hitachi S5200 field-emission microscope. For SEM characterization, a drop of DNA or DNA/AuNPs solution was spread on a stub; the solvent was removed and then used. The samples were examined without specific manipulation.

### 2.6. Solubility Measurements

The solubilities of pyren-1-carboxaldehyde in solutions containing gold nanoparticles were measured by agitating a generous excess of solid with the appropriate solution in a thermostated (298.2 K) vessel. After waiting a long time for undissolved solids to settle, an aliquot of the saturated solution was removed using a prethermostated pipette and the solution was diluted as necessary. Concentrations were measured spectrofluorimetrically in terms of previous calibration of the fluorescence of the pyren-1-carboxaldehyde at 5% ethanol with respect to the probe concentration. AuNPs concentrations corresponding to solubility measurements are given in Table 2.

## 3. Results and Discussion

Tiopronin-protected gold clusters are alkanethiolate nanoparticles in which the presence of a carboxylic and amino group of the tiopronin allows to modify the charge of the nanoparticle as a function of the pH of the medium. As a consequence, this nanosystem can be used positively or negatively charged. This colloid system also offers potential advantages due to the stability conferred by the attachment of thiol groups, high solubility in water, facile characterization, and functionalization. In this sense, the possibility of functionalizing the carboxylic group with small oligopeptides can be taken into account [36]. It is important to note that all experiments in this paper were carried out at pH = 6. According to the pKa value of the tiopronin bound to the gold cluster, pKa ≈ 5.6, Au@tiopronin nanoparticles are mostly neutral and hydrophilic [13, 35]. Therefore, the binding of tiopronin nanoparticles can be expected to occur principally through the formation of stable hydrogen bonding between the hydrophilic groups of the tiopronin and the DNA bases [13], and the strength of this interaction could be modified as a function of the media pH. In order to provide evidences of the interaction of tiopronin-protected gold nanoparticles with DNA, different techniques were employed.

**Figure 1:**
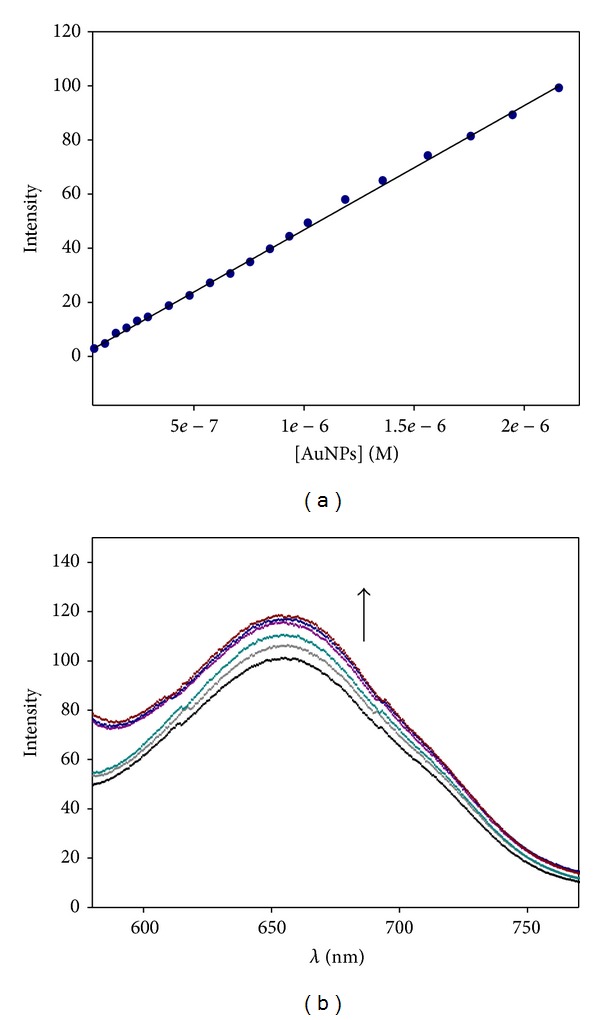
(a) Plot of fluorescence intensity at 656 nm of Au@tiopronin versus [AuNPs], in the absence of DNA. (b) Fluorescence spectra of AuNPs/DNA system recorded for increasing concentrations of DNA in 5% of ethanol. [AuNPs] = 1.5 × 10^−6^ M; [DNA] = 0 M (bottom); [DNA] = 7 × 10^−5^ M (top).

It is known that small gold nanoparticles are able to emit fluorescence [37]. Increasing amount of DNA was added to a solution containing a fixed nanoparticle concentration ([AuNPs] = 1.5 × 10^−6^ M) and the changes on the fluorescence spectra were measured. In order to know if the nanoparticle aggregates, the linearity of the Beer-Lambert law for solutions containing various nanoparticles concentrations was tested. The inset in Figure 1(a) shows that at low AuNPs concentrations (<2.16 *μ*M) the fluorescence intensity changes linearly with concentration, while at higher concentrations the Beer-Lambert law does not work, possibly due to a self-quenching effect produced by the Au@tiopronin. We must notice the importance of the small core size of the metal nanoparticle in relation to its luminescence properties. This feature is particularly of interest for water-soluble AuNPs having tiopronin thiolate monolayers, smaller than 2 nm. T. Huang et al. have studied the emission from tiopronin-AuNPs with different core sizes (1.8, 2.2, 3.1, and 3.9 nm) [37]. They found that only for the smallest nanoparticles (1.8 nm core size), luminescence was observed (*λ*_em_ = 770 nm). The luminescence maximum shifts to lower energy with increasing core size [38, 39]. In that sense, the results in Figure 1(b) obtained for our 1.4 nm AuNPs (*λ*_em_ = 656 nm) are in good agreement with the paper of Huang and Murray. On the other hand, as can be seen in Figure 1, the intensity of emission can be modified by the presence of DNA. In fact, an increase in the fluorescence emission at 656 nm was observed with increasing DNA concentration. Similar behaviour was observed for larger gold Au@tiopronin nanoparticles of 1.8 nm core size. However, one or two ethidium thiolate ligands are needed to be inserted into the Au@tiopronin/ethidium nanoparticles in order to confer better luminescent properties [29].

**Figure 2:**
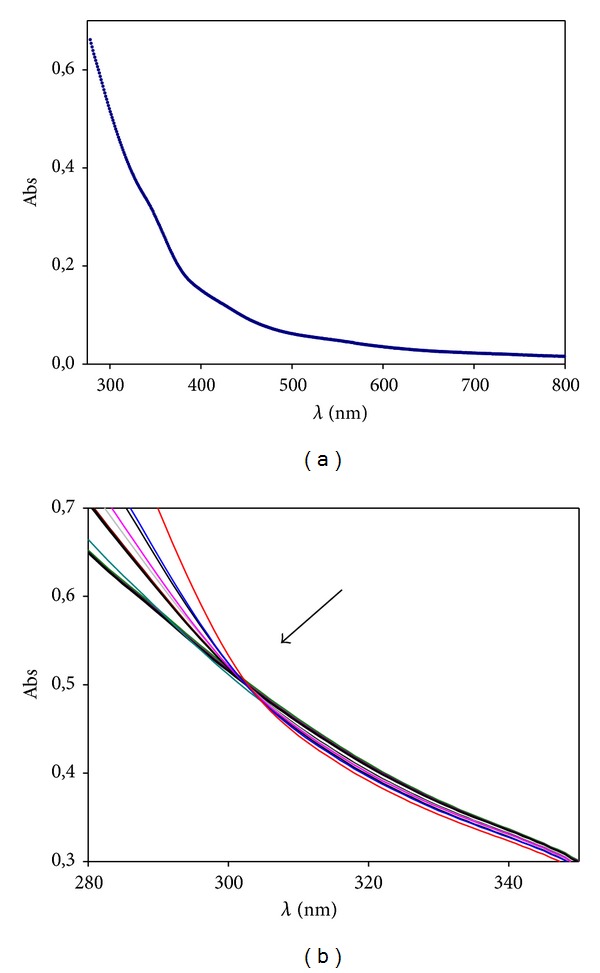
(a) Absorption spectrum of the Au@tiopronin. (b) Absorbance titration of AuNPs/DNA system recorded for increasing concentrations of DNA. [AuNPs] = 1.58 × 10^−6^ M; [DNA] = 0 M (top); [DNA] = 1 × 10^−4^ M (bottom).

With regard to UV-vis technique, Figure 2(b) shows the changes of the surface plasmon band (SPB) of colloidal gold as increasing amounts of DNA were added to the solution. Despite the small size of the nanoparticle, the SPB is almost nondetectable; in Figure 2 it can be observed how spectra are modified upon the binding of DNA. Moreover, the isosbestic point about 308 nm gives support to the DNA/Au@tiopronin complex formation.

**Figure 3:**
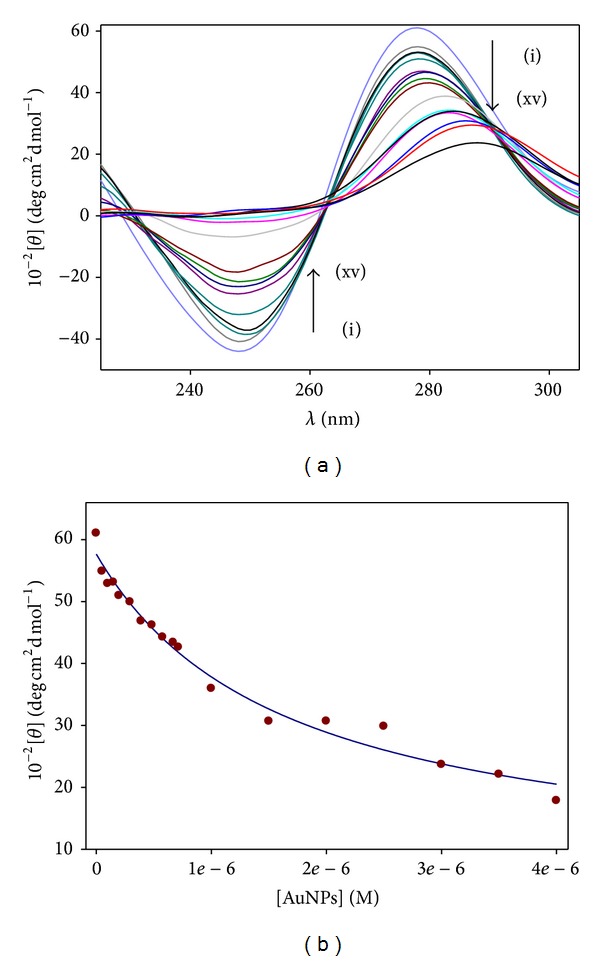
(a) CD titration of AuNPs with DNA in water. [DNA] = 1 × 10^−4^ M. Curves from (i) to (xv) correspond to 0, 0.15, 0.29, 0.39, 0.58, 0.71, 1.00, 1.50, 2.00, and 4.00 *μ*M of AuNPs. (b) Plot of the molar ellipticity experimental data versus AuNPs concentrations. Symbols (∙) are experimental data and line is the best fit using (2).

These two conventional spectroscopy techniques provide evidence of interaction. Unfortunately, due to the small changes registered on spectra as the titration occurs, these techniques are not appropriate to quantify the binding constant of the Au@tiopronin/DNA interaction. Alternatively, we found that the CD technique provides a convenient method to quantify this interaction. The determination of the free energy of binding, that is, the free energy for the DNA/AuNPs complex formation, has been carried out following two different approaches. The first one is based on the measurement of the changes of DNA molar ellipticity in the presence of AuNPs. The second approach is based on the use of a fluorescent probe, pyrene-1-carboxaldehyde. In both cases, the starting point has been a two-state model. DNA-AuNPs interaction causes a conformational change in the DNA structure during the course of the binding, which can be followed by the circular dichroism technique (see Figure 3). As is known, the backbone conformation of DNA shows a CD spectrum characteristic of the right-handed B form in the UV region (220–320 nm). Structure alterations of the DNA caused by its interaction with ligands are reflected in changes in this intrinsic CD spectrum. In Figure 3, the CD spectrum of free DNA has a positive peak at 279 nm and a negative peak at 247 nm which corresponds to B-DNA form. These bands are caused by stacking interactions between the bases and the helical suprastructure of the polynucleotide that provide an asymmetric environment for the bases [40]. In the same figure, an example of CD titration in water can be observed which clearly demonstrates that the helical conformation is not maintained.

Upon the addition of the gold clusters to the DNA solution, the molar ellipticity decreases at approximately 279 nm; meanwhile, it increases at approximately 247 nm. These changes are coupled with a shift in the maximum wavelength of the positive band, as can be seen in Figure 3(a), indicating partial denaturation of double strand [41, 42].

**Figure 4:**
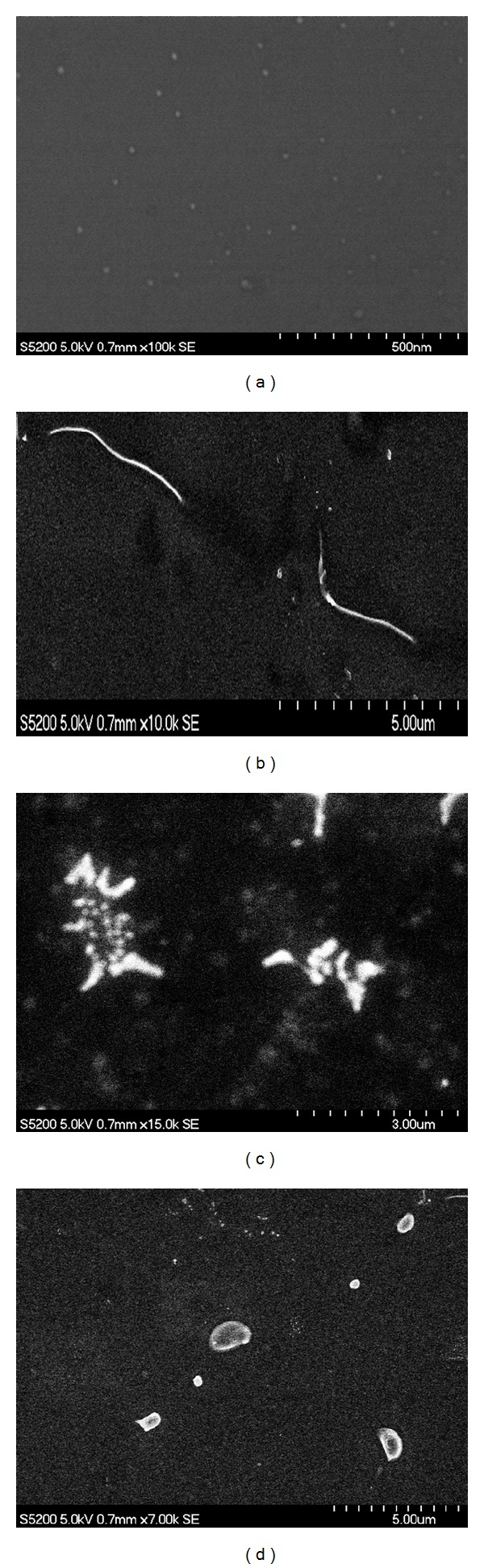
SEM images of CT-DNA at different concentrations of AuNPs in the presence of [NaCl] = 0.015 M. (a) [AuNPs] = 1 × 10^−6^ M; (b) [DNA] = 2 × 10^−6^ M; (c) [DNA] = 2.5 × 10^−6^, [AuNPs] = 2.5 × 10^−7^ M; (d) [DNA] = 2 × 10^−6^ M, [AuNPs] = 1 × 10^−6^ M.

Further information about what kind of DNA conformational changes being induced by Au@tiopronin nanoparticles has been obtained by means of the SEM technique. Typical images of CT-DNA in the absence (Figure 4(b)) and in the presence (Figures 4(c) and 4(d)) of different amount of gold nanoparticles are given in Figure 4. It is clear from these images that gold nanoparticles induce a compaction of DNA molecules. It can also be seen in the figure that the degree of compaction increases with the concentration of nanoparticles.

Based on CD spectrum changes as Au@tiopronin nanoparticles were added to the DNA solution, it was possible to quantify the binding constant of the interaction.

**Scheme 1:**
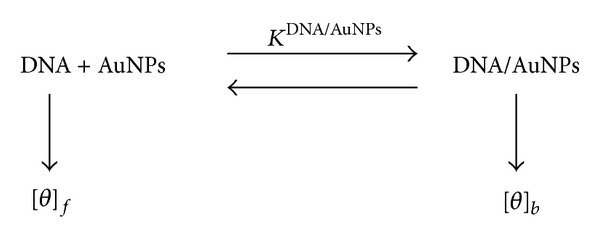


According to a two-state model, changes on the DNA molar ellipticity should be the consequence of the distribution of the gold cluster in the bulk (water) and on the DNA surface (see Scheme 1).

According to this model, if the DNA and Au@tiopronin nanoparticles constitute a complex, at the equilibrium there will be two populations of DNA: free and bound to the nanoparticle:

(1) $$\begin{array}{c} \left[\text{DNA}\right]=\frac{1}{1+K^{\text{DNA}/\text{AuNPs}}\left[\text{AuNPs}\right]}{\left[\text{DNA}\right]}_{0},\\ \left[\text{DNA}/\text{AuNPs}\right]=\frac{K^{\text{DNA}/\text{AuNPs}}\left[\text{AuNPs}\right]}{1+K^{\text{DNA}/\text{AuNPs}}\left[\text{AuNPs}\right]}{\left[\text{DNA}\right]}_{0}, \end{array}$$

where [DNA]_0_ is the total concentration of DNA, such that [DNA]_0_ = [DNA] + [DNA/AuNPs]. Accordingly, the observed molar ellipticity, [*θ*], would be given by

(2) $$\begin{array}{c} \left[\theta \right]=\frac{{\left[\theta \right]}_{f}+{\left[\theta \right]}_{b}K^{\text{DNA}/\text{AuNPs}}\left[\text{AuNPs}\right]}{1+K^{\text{DNA}/\text{AuNPs}}\left[\text{AuNPs}\right]}. \end{array}$$

Thus, *K* ^DNA/AuNPs^ can be obtained from the variations of [*θ*]_279 nm_ caused by the changes of [AuNPs].  Figure 3(b) gives these variations. Consequently, by fitting the experimental values of [*θ*] to (2), a value of *K* ^DNA/AuNPs^ = 6.4 × 10^5^ M^−1^ was obtained in water.

Similar experiments were carried out in the presence of different NaCl concentrations. The results (values of *K* ^DNA/AuNPs^) of these experiments are given in Table 1.

As can be seen in this table, the binding of DNA with AuNPs is somewhat sensitive to the ionic strength. This fact could be indicative of a slight electrostatic character of the binding. Therefore, the free energy of binding can be written as the sum of two contributions: (i) an electrostatic-potential-independent contribution, Δ*G*_nel_ (nonelectrostatic or intrinsic), and (ii) an electrostatic-potential-dependent contribution, Δ*G*_el_ (electrostatic). This separation has been discussed extensively in [43–47]:

(3) $$\begin{array}{c} \Delta G=\Delta G_{\text{nel}}+\Delta G_{\text{el}}. \end{array}$$

Thus, the free energy of binding Δ*G* can be written as a sum of two contributions: a nonelectrostatic contribution, Δ*G*_nel_, and an electrostatic one, Δ*G*_el_, which implies

(4) $$\begin{array}{c} K^{\text{DNA}/\text{AuNPs}}=K_{\text{nel}}K_{\text{el}}. \end{array}$$

In order to separate these contributions, we used Lippard's equation. According to Howe-Grant and Lippard [47], log *K*_el_ is proportional to −log[Na^+^]; that is,

(5) $$\begin{array}{c} \log K^{\text{DNA}/\text{AuNPs}}=\log K_{\text{nel}}-\beta \log\left[{\text{Na}}^{+}\right]. \end{array}$$

**Figure 5:**
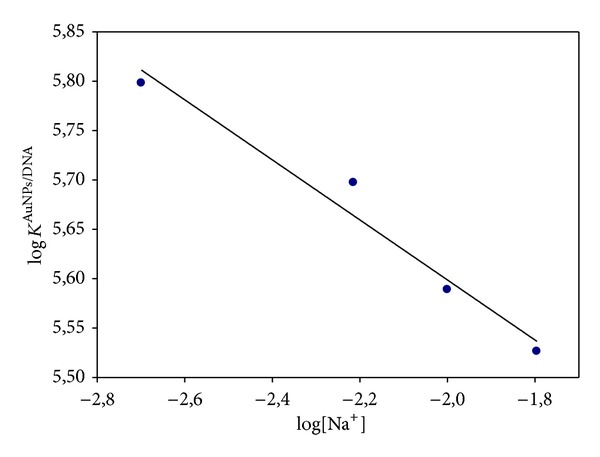
Plot of log *K* ^DNA/AuNPs^ versus log[Na^+^] (see (5)) for the DNA-AuNPs system.

The values of log⁡*K* ^AuNPs/DNA^ appearing in Table 1 are plotted in Figure 5. From the intercept, a value of log⁡*K*_nel_ = 4.98 was found, which gives a value of *K*_nel_ = 9.5 × 10^4^ M^−1^. That is, taking into account the values of *K* ^DNA/AuNPs^ appearing in Table 1, it can be established that the nonelectrostatic component of the binding free energy is about ~90% of the total free energy.

As mentioned previously, a second approach was used to obtain *K* ^DNA/AuNPs^. This approach is based on the use of a fluorescent intercalator pyrene-1-carboxaldehyde [48]. This procedure has been used by one of the previous authors to measure the binding constant of surfactant to DNA [11]. 1-PyCHO is a good probe for sensing polarity changes. This circumstance arises from the existence of two excited states that are close in energy: a fluorescent state and a “dark” state whose relative populations depend on the medium polarity. As a matter of fact, in the presence of DNA, we observed a diminution in the fluorescent intensity of 1-PyCHO [10, 49]. The magnitude of these changes in the intensity depends also on the association degree of the gold nanoparticle with DNA. This circumstance allows us to determine the binding constants of 1-PyCHO to DNA in the presence of different Au@tiopronin nanoparticles concentrations by using the Hildebrand-Benesi equation [50].

**Figure 6:**
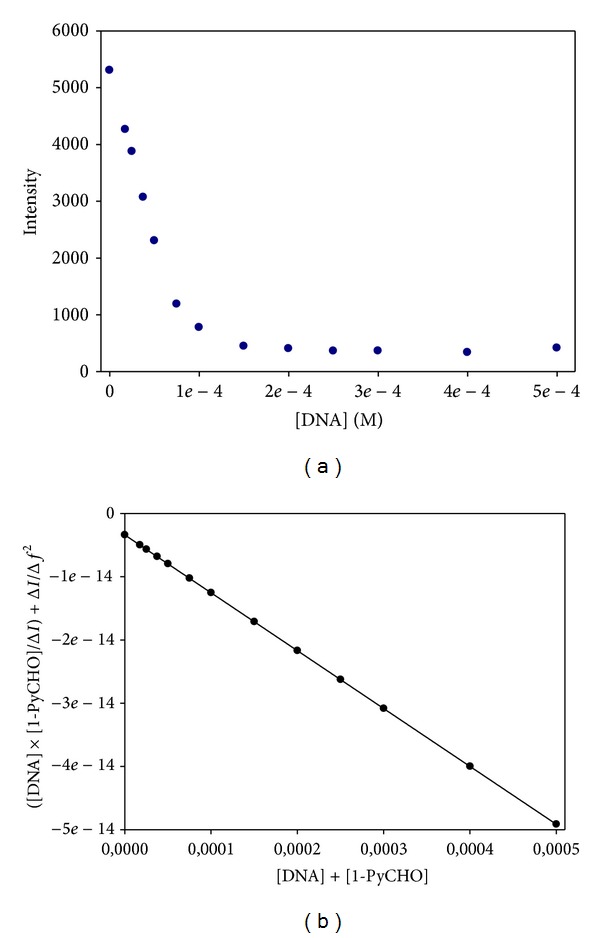
(a) Fluorescence titration (*λ*_em_ = 458 nm) of 1-PyCHO with DNA in the presence of Au@tiopronin nanoparticles. ([1-PyCHO] = 5 × 10^−7^ M; [AuNPs] = 6 × 10^−7^ ****M; [DNA] = 0–5 × 10^−4^ M, given in base pairs). (b) Fit of the data to Hildebrand-Benesi model.

We performed experiments in which the fluorescence emission of 1-PyCHO was measured in the presence of a fixed concentration of Au@tiopronin and variable DNA concentrations. A typical example of binding isotherm obtained from these titrations is given in Figure 6, and plot shows that the binding saturation has been attained at 1.5 × 10^−4^ M of DNA. Besides, as the AuNPs, concentration increases in each titration, the amount of DNA needed to attain saturation increases. The values of the binding constants of 1-PyCHO/DNA are given in Table 2.

As can be seen, these binding constants depend on the nanoparticle concentration, that is, on the proportion of DNA free and bound to the Au@tiopronin nanoparticles (see (1)). From these values of *K* ^1-PYCHO/DNA^, those of Δ*G* = −*RT* × ln⁡(*K* ^1-PYCHO/DNA^) were obtained. However, the values of Δ*G* represent the affinity of 1-PyCHO for the binding to DNA *relative to the solutions where the free pyrene-1-carboxaldehyde is present*. Obviously, Δ*G* cannot be compared directly because these solutions are different. However, solubility measurements in Table 2 allow us to compare Δ*G* once they have been corrected, taking into account the differences in free energy of the probe in the solutions due to the presence of tiopronin gold nanoparticles. Thus, if *S*_0_ is the solubility in the absence of Au@tiopronin and *S* in the presence of a given concentration of the nanoparticle, this free energy, Δ*G*, is related to the activity coefficient of 1-PyCHO, and the latter to the solubility:

(6) $$\begin{array}{c} \Delta G=RT\ln\gamma_{1\text{-PyCHO}}=RT\ln\frac{S_{0}}{S}. \end{array}$$

In this way, the values of Δ*G* ^CORR^ appearing in Table 2 were obtained:

(7) $$\begin{array}{c} \Delta G^{\text{CORR}}=\Delta G-RT\ln\frac{S_{0}}{S}. \end{array}$$

It is interesting to note that Table 2 shows that the Δ*G* ^CORR^ values increase when Au@tiopronin concentrations do so. According to this fact, it must be inferred that gold nanoparticles modify DNA upon binding. Au@tiopronin nanoparticles induce a change in the DNA/AuNPs complex that makes it less efficient than free DNA in order to bind 1-PyCHO. The effect of gold nanoparticles on DNA/1-PyCHO system is to induce a structural change in the DNA, which will be the result of the structural modifications due to the gold core and the tiopronin tails of the nanoparticle. Therefore, two kinds of DNA will be in the solution, free and bound to Au@tiopronin, with different capacities to bind 1-PyCHO, in the sense that the corrected binding free energy of the dye will be different for each kind of DNA. It can be shown (see Supporting Information available online at http://dx.doi.org/10.1155/2014/143645) that the observed corrected free energies, Δ*G* ^CORR^, will be given by

(8) ΔGCORR =ΔG1CORR+ΔG2CORR(K2/K1)KDNA/AuNPs[AuNPs]1+(1+K2)(KDNA/AuNPs/K1)[AuNPs].

In this equation, Δ*G*_1_^CORR^ is the corrected free energy of binding of 1-PyCHO with free DNA, that is, to DNA without Au@tiopronin; meanwhile, Δ*G*_2_^CORR^ is the same but when the DNA is bound to the gold nanoparticles, AuNPs; *K*_1_ is the equilibrium binding constant 1-PyCHO/DNA in the absence of Au@tiopronin and *K*_2_ is the equilibrium binding constant 1-PyCHO/DNA in the saturation limit of AuNPs. Finally, *K* ^DNA/AuNPs^ is the equilibrium constant for the binding of the Au@tiopronin with DNA (see Scheme 1). In other words, changes in Δ*G* ^CORR^ can be interpreted using a two-state model of the DNA/AuNPs binding.

**Figure 7:**
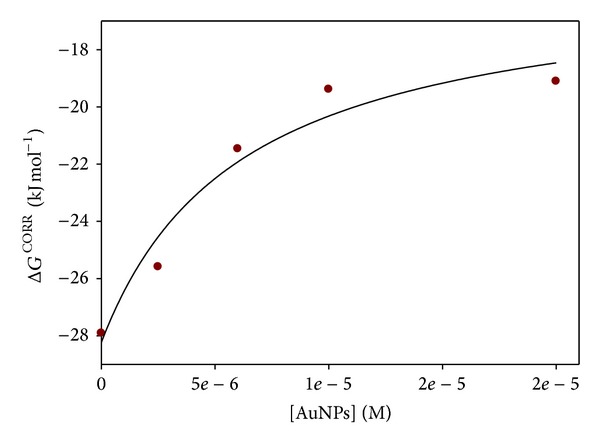
Corrected free energy of 1-PyCHO/AuNPs interaction, Δ*G* ^CORR^, versus [AuNPs], best fit to (8).

Figure 7 corresponds to the fit of data in Table 2 to (8). A value of *K* ^DNA/AuNPs^ = 5.9 × 10^5^ (M^−1^) was obtained, with *K*_2_ = 2.2 × 10^4^ and *K*_1_ = 8.1 × 10^4^ M^−1^. The agreement of this value with the corresponding one obtained through CD measurements supports our simpler methodology based on the two-state model.

## 4. Conclusions

The interaction of gold nanoparticles capped with tiopronin of 1.4 nm core size with DNA was investigated by using different techniques: CD, SEM, UV-vis, and fluorescence spectroscopy. A simple methodology to quantify the free energy of AuNPs/DNA binding has been proposed based on CD spectral change during AuNPs titration. A complementary study using pyrene-1-carboxaldehyde as a fluorescence probe supports the thermodynamic results obtained. Our results in the presence of different sodium chloride concentrations revealed that the AuNPs/DNA binding is fundamentally of nonelectrostatic character. The results provide an alternative method to quantify binding free energy of AuNPs/DNA-like systems when small changes in the titration are registered using conventional spectroscopic techniques.

## Supplementary Material

Detailed derivation of equation (8) and the model for evaluation of DNA-AuNPs equilibrium binding constants in the presence of two ligands that support the discussions in this manuscript.

## Figures and Tables

**Table 1:** Binding constants (*K* ^DNA/AuNPs^) and the free energy of binding (Δ*G* ^DNA/AuNPs^) corresponding to DNA/AuNPs interaction at different NaCl concentrations.

[NaCl]/M	10^5^ *K* ^DNA/AuNPs^/M^−1^	Δ*G* ^DNA/AuNPs^/kJmol^−1^
0.000	6.40	−33.12
0.002	6.34	−33.10
0.006	4.98	−32.50
0.010	3.88	−31.88
0.015	3.34	−31.51

**Table 2:** Solubilities (*S*), equilibrium binding constants (*K* ^1-PyCHO/DNA^), and the corrected free energy (Δ*G* ^CORR^) corresponding to 1-PyCHO/DNA interaction at different Au@tiopronin concentrations.

10^−6^ [AuNPs]/M	*S*/M	10^−4^ *K* ^1-PyCHO/DNA^/M^−1^	(Δ*G* ^CORR^)/kJmol^−1^
0.0	1.21 × 10^−6^	78.0	−27.91
2.5	3.02 × 10^−6^	77.0	−25.59
6.0	1.21 × 10^−5^	58.1	−21.47
10.0	2.41 × 10^−5^	49.8	−19.39
20.0	2.49 × 10^−5^	46.0	−19.11
