# Supplementary material for: Noncovalent Interactions of Tiopronin-Protected Gold Nanoparticles with DNA: Two Methods to Quantify Free Energy of Binding
Source: ScientificWorldJournal. 2014 Jan 22;2014:143645. doi: 10.1155/2014/143645 (PMC3920862; doi:10.1155/2014/143645)
Supplement: Supplementary file 1 — Detailed derivation of equation (8) and the model for evaluation of DNA-AuNPs equilibrium binding constants in the presence of two ligands that support the discussions in this manuscript. [file 143645.f1.pdf]

SUPPORTING INFORMATION.

The two state model for DNA conformational changes in the presence of two ligands: derivation of equation (10).

Assuming that gold colloid nanoparticles interact directly with DNA promoting a conformational change in the biomolecule, there are two kinds of DNA in the solution: free (DNA_1) and bound to the nanocluster (DNA_2). These forms of DNA present different affinity to interact with the fluorescent probe, pyrene-1carboxaldehyde. Thus, in the colloidal gold system, containing DNA and the 1-PyCHO probe, there are at least three equilibria implicated. One of them, the DNA/AuNPs equilibrium binding, appears as the DNA conformation changes from the state 1, where DNA is free in solution (DNA_1), to the state 2 in which DNA is stabilized on the nanoparticle surface (DNA_2):

$$\mathrm{DNA_1 + AuNPs \underset{}{\overset{K^{DNA/AuNPs}}{\rightleftarrows}} DNA_2} \qquad (A_1)$$

$$\mathrm{K^{DNA/AuNPs} = \frac{[DNA_2]}{[DNA_1][AuNPs]}} \qquad (A_2)$$

The remaining equilibria correspond to the binding of the probe with DNA in the two different DNA conformations that are present in the AuNPs/DNA system. One of them corresponds to the binding 1-PyCHO/DNA_1 where the biomolecule is free of AuNPs. This binding is represented by equations A_3-A_4:

$$\mathrm{DNA_1 + 1 - PyCHO \overset{K_1}{\rightleftarrows} DNA_1/1\text{-}PyCHO} \qquad (A_3)$$

$$\mathrm{K_1 = \frac{[DNA_1/1\text{-}PyCHO]}{[DNA_1][1\text{-}PyCHO]}} \qquad (A_4)$$

On the other hand, the corresponding equilibrium for the binding of the probe to the DNA in state 2 (linked to gold nanoparticles) is given by equations A_5-A_6:

$$DNA_2 + 1 - PyCHO \underset{}{\overset{K_2}{\rightleftarrows}} DNA_2/1\text{-}PyCHO \qquad (A_5)$$

$$K_2 = \frac{[DNA_2/1\text{-}PyCHO]}{[DNA_2][1\text{-}PyCHO]} \qquad (A_6)$$

In general, whatever property of interest of the system you consider, (P), the measured value quantity of interest, ($\langle P \rangle$), is the sum of the fractional contributions of each state:

$$\langle P \rangle = \sum X_i P_i \qquad (A_7)$$

$$\langle P \rangle = P_1X_1 + P_2X_2 + P_3X_3 \qquad (A_8)$$

where $\langle P \rangle$ is the average value of a general property designed as "P" and X_i is the contribution of "P" to the state "i". Regardless of the number of states, the set of numbers $\{X_i\}$ defines the distribution of states. In the present paper, the distribution corresponds to the fraction of 1-PyCHO that binds to DNA in state 1, DNA in the state 2, and the fraction of free 1-PyCHO. Considering that the fractional contribution of the free probe is meaningless compared to the 1-PyCHO bound (in our working conditions the intercalation process is almost complete), its fractional contribution can be neglected and the equation A_8 can be simplified. Taking into account the equilibria described previously, the fractional contribution to the state 1 and 2 is given by:

$$X_1 = \frac{[DNA_1]}{[DNA]} = \frac{K_1[DNA_1]}{1 + K_1[DNA_1] + K_2[DNA_2]} \qquad (A_9)$$

$$X_2 = \frac{[DNA_2]}{[DNA]} = \frac{K_2[DNA_2]}{1 + K_1[DNA_1] + K_2[DNA_2]} \qquad (A_{10})$$

$$X_1 + X_2 + X_3(\approx 0) = 1 \qquad (A_{11})$$

Combining the expressions A_9 and A_{10} in the equation A_8 we have:

$$\langle P \rangle = \frac{P_1 K_1 [DNA_1] + P_2 K_2 [DNA_2]}{1 + K_1 [DNA_1] + K_2 [DNA_2]} \qquad (A_{12})$$

Then, considering the equilibrium DNA/AuNPs described by the equations A_1-A_2, the DNA concentration in state 1 and 2 will be given by:

$$[DNA_1] = \frac{1}{1 + K^{DNA/AuNPs}[AuNPs]}[DNA] \qquad (A_{13})$$

$$[DNA_2] = \frac{K^{DNA/AuNPs}[AuNPs]}{1 + K^{DNA/AuNPs}[AuNPs]}[DNA] \qquad (A_{14})$$

The following expression is given introducing these DNA concentrations ($[DNA_1]$ and $[DNA_2]$) into the equation (A_{12}) and reorganizing:

$$\langle P \rangle = \frac{P_1 + P_2 \frac{K_2}{K_1} K^{DNA/AuNPs}[AuNPs]}{1 + (1 + K_2)\frac{K^{DNA/AuNPs}}{K_1}[AuNPs]} \qquad (A_{15})$$

This equation relates the changes of any property of the system with the changes in substrate concentrations ([AuNPs] in our case). Considering the corrected free energy of binding DNA/1-PyCHO, ΔG^{CORR}, as a property of interest we obtain the final expression (eq. (10) in the text):

$$\langle \Delta G^{CORR} \rangle = \frac{\Delta G_1^{CORR} + \Delta G_2^{CORR} \frac{K_2}{K_1} K^{DNA/AuNPs}[AuNPs]}{1 + (1 + K_2)\frac{K^{DNA/AuNPs}}{K_1}[AuNPs]} \qquad (A_{16})$$
